# Inflammation-related biomarkers and berberine therapy in post-stroke depression: evidence from bioinformatics, machine learning, and experimental validation

**DOI:** 10.3389/fnins.2025.1684297

**Published:** 2025-10-14

**Authors:** Wei Liu, Ruheng Wei, Jingya Xu, Zhilong Liu, Yulai Li

**Affiliations:** ^1^Graduate School of Hebei North University, Zhangjiakou, Hebei, China; ^2^Department of Curative Diseases, Langfang Hospital of Traditional Chinese Medicine, Langfang, Hebei, China; ^3^Jingxing County Hospital, Shijiazhuang, Hebei, China; ^4^Cangzhou Hospital of Integrated Traditional Chinese and Western Medicine, Cangzhou, Hebei, China

**Keywords:** post-stroke depression, inflammation, traditional Chinese medicine, bioinformatics, machine learning

## Abstract

**Objective:**

Post-stroke depression (PSD), a common neuropsychiatric complication, significantly hinders stroke recovery and quality of life. Given the established role of inflammation in the pathogenesis of PSD, this study aimed to identify key inflammation-related genes and pathways using bioinformatics and machine learning and further evaluate the protective effects of traditional Chinese medicine (TCM) monomer compounds.

**Methods:**

PSD-related datasets (GSE16561, GSE98793) were obtained from the Gene Expression Omnibus (GEO). Differentially expressed genes (DEGs) were identified using the limma package, followed by functional enrichment analysis with Gene Ontology (GO) and Kyoto Encyclopedia of Genes and Genomes (KEGG). Three machine learning algorithms—random forest, support vector machine-recursive feature elimination (SVM-RFE), and least absolute shrinkage and selection operator (LASSO)—were applied to screen inflammation-related hub genes. Immune cell infiltration was analyzed using single-sample gene set enrichment analysis (ssGSEA). Candidate TCM compounds were explored via the Coremine Medical database. A PSD rat model was established to validate hub gene expression and to assess the efficacy of berberine (BBR).

**Results:**

Analysis identified 35 inflammation-related DEGs (IDEGs) significantly enriched in immunological processes, including malaria pathogenesis, NETosis, innate immune deficiencies, Rap1 signaling, and IL-17 cascades. The integration of machine learning pinpointed TLR2 and CYP1B1 as core hub genes, demonstrating robust diagnostic performance in external validation. Molecular docking suggested a strong binding affinity between the TCM compound BBR and TLR2/CYP1B1 proteins. PSD rats exhibited prolonged immobility in forced swim/tail suspension tests and decreased sucrose preference versus controls, alongside neuronal damage, edema, and inflammatory infiltration (HE staining). BBR treatment reversed these behavioral deficits and pathological changes. Western blot analysis confirmed elevated TLR2 and CYP1B1 expression in PSD rats, significantly downregulated by BBR. Enzyme-linked immunosorbent assay (ELISA) showed increased serum IL-1β, IL-6, and TNF-*α* levels in PSD, which BBR effectively reduced.

**Conclusion:**

This study identifies TLR2 and CYP1B1 as core inflammation-related genes in PSD. BBR demonstrates therapeutic efficacy as an active monomer compound against PSD, likely mediated through downregulating TLR2 and CYP1B1 expression, consequently diminishing the concentrations of pro-inflammatory mediators (IL-1β, IL-6, TNF-*α*) that mediate cerebroprotective actions.

## 1 Introduction

Globally, stroke persists as the second most prevalent mortality factor, characterized by high rates of disability and mortality, often resulting in severe cognitive impairment and physical disability (GBD 2019 Stroke Collaborators, 2021). Post-stroke depression (PSD), the most frequently occurring neurological/psychiatric disorder developing after cerebrovascular events, exhibits a cumulative incidence of 39–55% within 5 years post-event (Villa et al., 2018). PSD not only manifests as anhedonia, attentional deficits, and related symptoms but also impedes functional rehabilitation and elevates mortality risk (Robinson and Jorge, 2016). However, the insidious onset of PSD frequently leads to misdiagnosis or underdiagnosis (Lai and McCullough, 2019). The clinical diagnosis of PSD is further compounded by the absence of reliable diagnostic biomarkers. Therefore, identifying novel biomarkers for PSD has significant clinical implications for early diagnosis and targeted therapeutic interventions.

The pathogenesis of PSD remains incompletely elucidated, involving multifactorial mechanisms such as hypothalamic–pituitary–adrenal (HPA) axis dysfunction, the neurotrophic hypothesis, neurotransmitter alterations, and immune-inflammatory responses (Feng et al., 2024). Previous studies indicated that inflammatory cascades critically contribute to PSD development. Cerebral ischemia triggers activation of damage-associated molecular patterns (DAMPs), which, upon binding to specific receptors, initiate excessive release of proinflammatory cytokines (Chen and Nuñez, 2010). These cytokines promote depression by damaging stress-vulnerable brain regions, including the hippocampus and accelerating depletion of serotonin (5-hydroxytryptamine, 5-HT) (Gulyaeva, 2019b; Robinson et al., 1984). Clinically, elevated serum levels of interleukin-1β (IL-1β), interleukin-6 (IL-6), and tumor necrosis factor-*α* (TNF-α) at hospital admission correlate with significantly higher PSD risk in stroke patients (Li et al., 2022; Chen et al., 2024).

This research aimed to identify critical genetic markers linked to the pathogenesis of PSD through bioinformatics analysis (Wei et al., 2022; Liu et al., 2020), explore their underlying mechanisms, and predict potential therapeutic compounds. By analyzing PSD-related datasets from the GEO database, we identified differentially expressed inflammation-related genes (IDEGs) and performed functional enrichment analysis. Machine learning algorithms were integrated to screen two hub genes (TLR2 and CYP1B1).

Analysis of GEO datasets revealed TLR2 and CYP1B1 as key inflammation-related hub genes in PSD.

Despite the increasing recognition of the inflammatory basis of PSD, notable gaps remain in understanding the specific molecular biomarkers that drive its onset and progression. Existing studies have primarily focused on classical mechanisms, including hypothalamic–pituitary–adrenal axis dysfunction, neurotransmitter imbalance, and general immune activation, yet reliable diagnostic markers for PSD are still lacking. Moreover, although traditional Chinese medicine (TCM) compounds have shown promising antidepressant and neuroprotective effects, their role in modulating inflammatory pathways in PSD remains underexplored. For instance, compounds such as baicalin, curcumin, and paeoniflorin have been reported to alleviate depressive-like behaviors by reducing neuroinflammation and oxidative stress, but systematic validation in the context of PSD is insufficient. Therefore, this research aimed to fill the gap by integrating bioinformatics and machine learning to identify inflammation-related hub genes in PSD and by further investigating the therapeutic potential of TCM monomers, particularly BBR, as targeted interventions for neuroinflammatory regulation.

Based on the two targets, we predicted and validated potential TCM compounds via molecular docking. Finally, experimental validation in PSD rat models confirmed both the functional relevance of these genes and their viability as therapeutic targets.

## 2 Materials and methods

### 2.1 Data acquisition and processing

Microarray datasets related to ischemic stroke (IS) and major depressive disorder (MDD) were retrieved from the Gene Expression Omnibus (GEO) database^1^ using the keywords “stroke” and “depression.” (accession: GSE16561 with 39 IS/34 control samples; GSE58294 containing 69 cardioembolic stroke samples/23 normal specimens).

The GSE98793 dataset comprised 128 MDD samples (including 64 patients with comorbid generalized anxiety disorder and 64 with non-anxious depression) alongside 64 demographically matched healthy controls. Per analytical protocol, anxiety-comorbid MDD cases were excluded, ultimately yielding a curated cohort of 64 non-comorbid depression patients and 64 demographically matched controls. GSE38206 consisted of nine major depressive episode samples and nine healthy controls. For validation purposes, the GSE58294 and GSE38206 datasets were designated as validation sets in this study.

Data processing was performed using R software (v4.3.2). Expression matrices were quantile-normalized with the “normalize Between Arrays” function from the “limma” package (Ritchie et al., 2015). Probe IDs were annotated to gene symbols using platform-specific mappings, with probes lacking gene symbols excluded and multi-probe genes consolidated by averaging expression values. Final expression matrices were log2(x + 1) transformed. Inflammation-related genes were retrieved from the GeneCards database^2^ using “inflammation” as the search term, yielding 16,200 genes for subsequent analysis.

### 2.2 Identification of PSD-related inflammatory genes

The “limma” R package was utilized to conduct differential gene expression analysis, with IS-related DEGs screened from the GSE16561 dataset applying cutoff criteria of absolute log2 fold change >0.5 and *p*-value <0.05. Similarly, DEGs associated with MDD were screened in the GSE98793 dataset with thresholds of |log2FC| > 0.25 and *p*-value < 0.05. Volcano plots and heatmaps visualizing DEG expression patterns were generated using ggplot2 and pheatmap packages. Overlapping DEGs associated with both IS and MDD pathogenesis were identified using the Venny online tool^3^. These shared DEGs were designated as key PSD-related genes for downstream analyses. Finally, inflammation-associated DEGs (IDEGs) specific to PSD were identified by intersecting these key genes with the curated inflammation-related gene set. To account for the distinct biological characteristics and sample sizes of the two datasets, different cutoff thresholds were applied when identifying DEGs. Specifically, for the ischemic stroke dataset (GSE16561), a more stringent threshold of $|\log_{2}\mathrm{FC}|>0.5$ was chosen to reduce potential false positives, given its relatively smaller sample size and higher inter-sample variability. In contrast, for the major depressive disorder dataset (GSE98793), a more lenient cutoff of $|\log_{2}\mathrm{FC}|>0.25$ was applied to capture a broader range of genes with modest but biologically relevant expression changes, considering the larger cohort and higher statistical power. Importantly, the reliability of the results was further confirmed by machine learning feature selection and external validation analyses. Thus, the differential thresholds do not compromise comparability but instead ensure a balance between sensitivity and specificity in identifying candidate PSD-related genes.

### 2.3 Functional enrichment analysis

To characterize the biological roles and molecular pathways of the IDEGs, we conducted Gene Ontology (GO) and Kyoto Encyclopedia of Genes and Genomes (KEGG) analyses implemented in the clusterProfiler R package (v4.0), applying a statistically significant cutoff of *p* < 0.05 (Subramanian et al., 2005). The GO annotation encompasses three domains, including biological processes (BP), molecular functions (MF), and cellular components (CC). KEGG analysis elucidated functional pathways and gene interactions. Results were visualized using ggplot2 through bar plots for enhanced interpretability.

### 2.4 Machine learning algorithms

Three established machine learning algorithms were employed to identify potential PSD diagnostic biomarkers: SVM-RFE, LASSO regression, and RF. A fixed random seed of 123 ensured reproducibility across datasets. SVM-RFE was implemented using the e1071 package to classify high-dimensional data into finite support vectors for dimensionality reduction (Cortes and Vapnik, 1995; Liu et al., 2025). Feature selection was performed via LASSO regression with the glmnet package (Tibshirani, 2018). The randomForest package (Ishwaran and Kogalur, 2010) enabled RF modeling to interpret feature correlations and interactions, visualizing the top 15 genes by importance ranking. Core genes with optimal PSD relevance were identified by intersecting consensus results from all three algorithms.

### 2.5 Diagnostic efficacy of biomarkers

External validation was performed using the IS dataset (GSE58294) and the MDD dataset (GSE38206). Receiver operating characteristic (ROC) curves were constructed with the pROC package. Box plots visualized diagnostic gene expression patterns in validation cohorts, and the area under the curve (AUC) was calculated to assess biomarker performance (Robin et al., 2011).

### 2.6 Immune cell infiltration

Single-sample gene set enrichment analysis ssGSEA was performed in R version 4.3.2 using the GSVA package. This quantified relative proportions of 28 immune cell types within gene expression matrices, characterizing immune infiltration landscapes across IS and MDD samples (Hänzelmann et al., 2013).

### 2.7 TCM prediction and molecular docking

The Coremine Medical database^4^ was queried to screen TCMs with potential therapeutic effects, using a significance threshold of *p* < 0.05. When candidate compounds exceeded a predefined threshold indicating an excessive number, further refinement was applied based on TCM theoretical principles and clinical practice standards. Active constituents of prioritized TCMs were retrieved from TCMSP^5^ using pharmacokinetic filters: oral bioavailability (OB) ≥ 30% and drug-likeness (DL) ≥ 0.18. Protein structures of key target genes were obtained in PDB format from the RCSB database^6^. Molecular docking was performed using CB-Dock2^7^, which features enhanced docking accuracy through integration with AutoDock Vina. This tool predicts ligand-binding cavities via curvature-based cavity detection, computes binding site coordinates, ranks poses by Vina scores (prioritizing high absolute binding affinity values), and provides interactive 3D visualization of binding conformations.

### 2.8 Experimental animals and grouping

Thirty adult male Sprague–Dawley (SD) rats (SPF grade, body weight 250–280 g) were obtained from Liaoning Changsheng Biotechnology Company (Animal Production License: SCXK(Liao)2020–0001). Experiments were conducted at the Neuroimmunology Laboratory of Cangzhou Hospital of Integrated Traditional Chinese and Western Medicine. Rats were randomly assigned to three groups (*n* = 10 per group): a Sham surgery group, a PSD model group, and a berberine (BBR) treatment group. The Sham surgery group underwent incision and suturing without vascular occlusion. The PSD model group received middle cerebral artery occlusion (MCAO) followed by chronic unpredictable mild stress (CUMS). The BBR group was administered BBR hydrochloride (50 mg/kg/day, Aladdin, B414323) orally for seven consecutive days after the PSD model was established. This study was approved and monitored by the Animal Ethics Committee of Cangzhou Hospital of Integrated Traditional Chinese and Western Medicine (Approval No. CZX2024134). The dosage of berberine hydrochloride (50 mg/kg/day) was selected based on previous studies demonstrating its neuroprotective and anti-inflammatory effects in rodent models of cerebral ischemia and depression-like behaviors (Mujtaba et al., 2022). Furthermore, this dose has been widely reported as safe and effective in preclinical studies, providing sufficient systemic exposure for assessing central pharmacological actions.

### 2.9 Modeling methodology

In the present study, the PSD model was established by combining middle cerebral artery occlusion (MCAO) with chronic unpredictable mild stress (CUMS). First, MCAO was used to induce ischemic stroke in rats, producing consistent neurological deficits that mimic the cerebrovascular pathology of stroke. One week after successful MCAO surgery, rats were subjected to a 4-week CUMS protocol to induce depression-like behaviors. The CUMS procedure included seven randomized stress modalities (tail pinching, 24-h food or water deprivation, physical immobilization, inverted light/dark cycle, foot shock, and forced cold-water swimming). One stressor was applied per day, and the sequence was randomized to avoid habituation. This combination of MCAO and CUMS has been widely validated to reproduce the core features of PSD, including both neurological impairment and depressive-like phenotypes. The PSD model was established by anesthetizing rats with intraperitoneal sodium pentobarbital (30–50 mg/kg). After exposing the left common carotid artery, the external carotid artery and its branches were carefully isolated and coagulated before advancing a blunt-tipped 3–0 nylon suture from the external carotid stump into the internal carotid artery lumen, advancing to the anterior cerebral artery to induce MCAO. After 90 min of occlusion, the suture was withdrawn for reperfusion. Rats recovered under infrared heating lamps, maintaining ambient temperature at 35 ± 2 °C, with rectal temperature regulated 37 ± 0.5 °C with an automated heating system.

Neurological deficits were assessed 24 h postoperatively using the Longa scoring system (Kong et al., 2017), selecting rats that scored ≥1 but less than 4 for subsequent procedures. Beginning on postoperative day 7, successful MCAO rats underwent a four-week course of CUMS. Depression-like behaviors were screened through forced swimming, tail suspension, and sucrose preference tests (Okaty et al., 2019). The CUMS protocol administered one randomly selected daily stimulus from seven modalities: tail pinching for 1 min, 24-h water/food restriction, 5-min physical immobilization, 24-h inverted photoperiod, 1-min foot shock, or 5-min 4 °C forced swim, with randomized sequencing to prevent consecutive duplicate stimuli. The success of the MCAO model was validated 24 h after surgery using the Longa neurological deficit scoring system. Only rats with scores ≥1 and <4 were included in subsequent PSD modeling to ensure consistency of ischemic injury severity before the induction of depression-like behaviors.

### 2.10 Sucrose preference test (SPT)

The sucrose preference test quantified anhedonia, a core feature of PSD, by measuring rats’ diminished responsiveness to reward. All rats were acclimated to 1% sucrose solution through initial 24-h exposure to two sucrose bottles, followed by 24-h exposure to one sucrose bottle and one water bottle. After 24 h of food and water deprivation, the formal test was conducted for 1 h. During testing, rats had free access to both 1% sucrose solution and plain water (200 mL each). Sucrose preference percentage was calculated as [sucrose solution consumption / (sucrose solution consumption + plain water consumption)] × 100%.

### 2.11 Tail suspension test (TST)

The TST assessed behavioral despair by immobilizing rats. The distal third of each rat’s tail was secured with adhesive tape to an elevated bracket, positioning the head 15 cm above the platform. The testing was video-recorded against a black background for over 6 min. Immobility was measured during the last 4-min interval using Smart 2.5 tracking software.

### 2.12 Forced swim test (FST)

The FST evaluates behavioral despair in experimental subjects. It was 24 h before formal testing that rats underwent a 5-min acclimation swim. Rats were immersed in water maintained at 23–25 °C, with body balance ensured, after which timing commenced. During the 6-min trial, immobility time, defined as the absence of movement in the trunk and limbs, was recorded specifically during the final 4 min.

### 2.13 Hematoxylin–eosin staining (HE)

Brain specimens were processed through standard histological preparation, including fixation, dehydration, clearing, and paraffin embedding, followed by 5 μm sectioning and hematoxylin–eosin staining, followed by sequential treatments: differentiation in 1% hydrochloric acid, bluing with ammonium hydroxide, eosin counterstaining, water rinsing, graded ethanol dehydration, baking, and mounting for microscopic histomorphological analysis. Quantitative assessment of neuronal death rate was performed in the hippocampal CA1 region of each rat. Neuronal death rate was calculated as the percentage of pyknotic or shrunken neurons among the total number of neurons in three randomly selected fields at the same magnification. All analyses were performed by two blinded observers using ImageJ software, and the results were averaged for statistical analysis.

### 2.14 Inflammatory cytokine detection by ELISA

Inflammatory cytokine levels, including TNF-*α*, IL-1β, and IL-6 in brain tissue, were measured using enzyme-linked immunosorbent assay (ELISA) kits from Solarbio, Beijing, China. Briefly, 100 mg of frozen brain tissue was homogenized in phosphate-buffered saline and centrifuged at 10000 r/min with a 10 cm rotor radius for 10 min. The resulting supernatant was collected for subsequent analysis following the manufacturer’s protocols.

### 2.15 Western blot (WB) analysis of TLR2 and CYP1B1 protein levels

Brain tissue lysates were prepared by homogenizing the samples in protease-containing lysis buffer, followed by centrifugation to collect supernatants. Total protein concentration was quantified before electrophoresis. Target protein bands were electrophoretically transferred onto PVDF membranes following gel excision.

After blocking, the membranes were incubated overnight at 4 °C with primary antibodies against TLR2 (1:1000, Abcam, EPR20303) and CYP1B1 (1:1000, Abcam, EPR14972). After secondary antibody incubation, protein signals were detected by chemiluminescence and quantified through grayscale analysis with ImageJ software.

### 2.16 Statistical analysis

All data were expressed as mean ± standard error of the mean (SEM). Statistical analyses were performed using one-way analysis of variance (ANOVA) followed by Tukey’s *post hoc* test for multiple group comparisons. Differences were considered statistically significant at *p* < 0.05. Graphs and statistical calculations were generated using GraphPad Prism version 9.0 (GraphPad Software, San Diego, CA, USA). The sample size was n = 10 rats per group for all behavioral tests, ELISA assays, H&E staining, and Western blot analyses.

## 3 Results

### 3.1 Screening of inflammation-related genes in PSD

**Figure 1:**
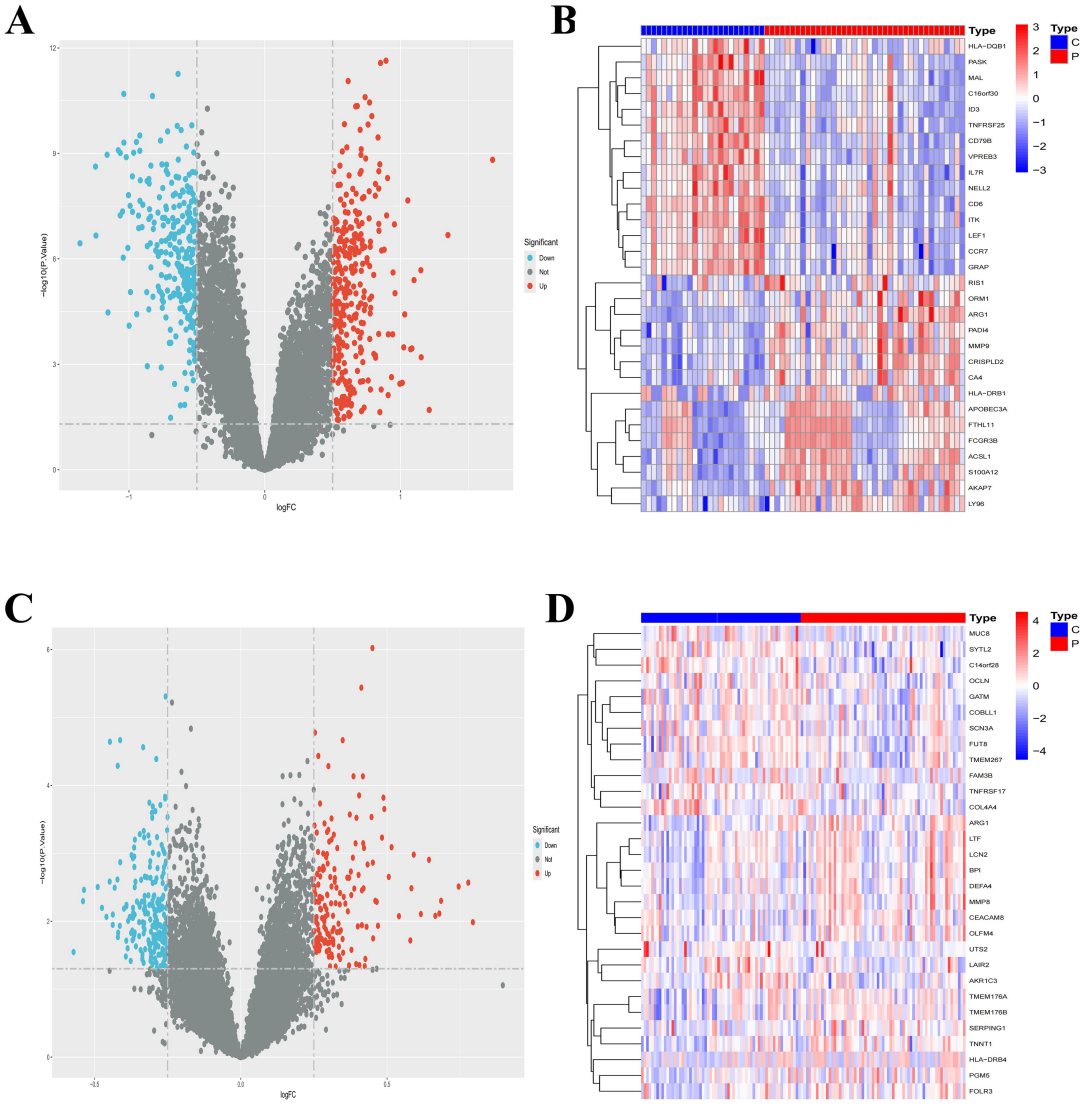
**(A)** Volcano plot of differentially expressed genes (DEGs) between ischemic stroke patients and controls in GSE16561. **(B)** Heatmap of DEGs in GSE16561. **(C)** Volcano plot of DEGs between major depressive disorder patients and controls in GSE98793. **(D)** Heatmap of DEGs in GSE98793.

Using a threshold of *p* < 0.05 and |log2FC| > 0.5, we identified 588 DEGs in the IS dataset GSE16561, comprising 307 upregulated and 281 downregulated genes. For the MDD dataset GSE98793, 411 DEGs were screened using significance criteria of p < 0.05 and absolute log2 fold change >0.25, including 169 upregulated and 242 downregulated genes. Volcano plots visualized the DEG distributions for both IS and MDD cohorts (Figures 1A,C). Heatmaps revealed the 15 most significantly upregulated and downregulated genes in each dataset (Figures 1B,D).

Intersection analysis using the Venny online tool (http://vip.sangerbox.com/) revealed 44 common DEGs between inflammation-related genes and the IS and MDD DEGs (Supplementary Figure 1). These overlapping DEGs were designated as key PSD-related genes for subsequent analysis. Further integration of these DEGs with established inflammation-associated genes yielded 35 core PSD inflammation-related genes for downstream investigation (Supplementary Figure 2).

### 3.2 Identification and enrichment analysis of PSD inflammation-related genes

**Figure 2:**
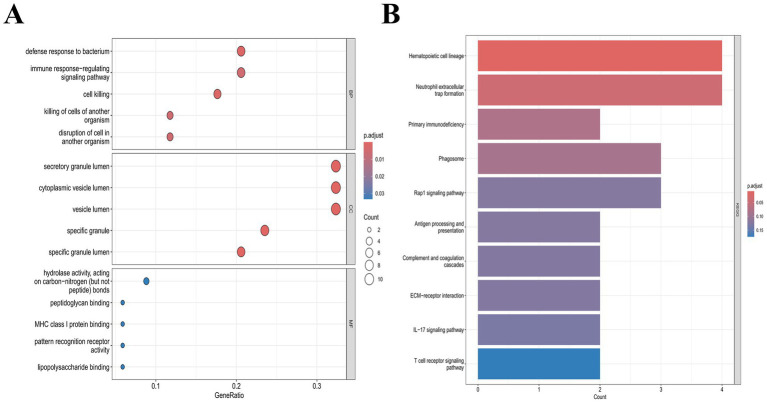
GO and KEGG enrichment analyses of PSD. **(A)** Shows a bubble plot depicting enrichment in three Gene Ontology categories: BP, CC, and MF. **(B)** Displays a bar chart illustrating the top 10 KEGG pathways significantly enriched in PSD.

GO and KEGG enrichment analyses were performed on these IDEGs. The GO results (Figure 2A) indicated that these IDEGs were primarily enriched in Biological Process (BP) terms related to defense response to bacterium, regulation of immune response, and cell killing; for Cellular Component (CC) they were mainly associated with the secretory granule lumen, cytoplasmic vesicle lumen, and vesicle lumen; and for Molecular Function (MF) they were significantly involved in pathways such as peptidoglycan binding, Toll-like receptor binding, and immunoglobulin binding. KEGG pathway analysis revealed that the IDEGs were predominantly enriched in pathways including malaria, neutrophil extracellular trap formation, primary immunodeficiency, Rap1 signaling pathway, IL-17 signaling pathway, and T cell receptor signaling pathway (Figure 2B).

### 3.3 Identification of potential hub genes using machine learning algorithms

**Figure 3:**
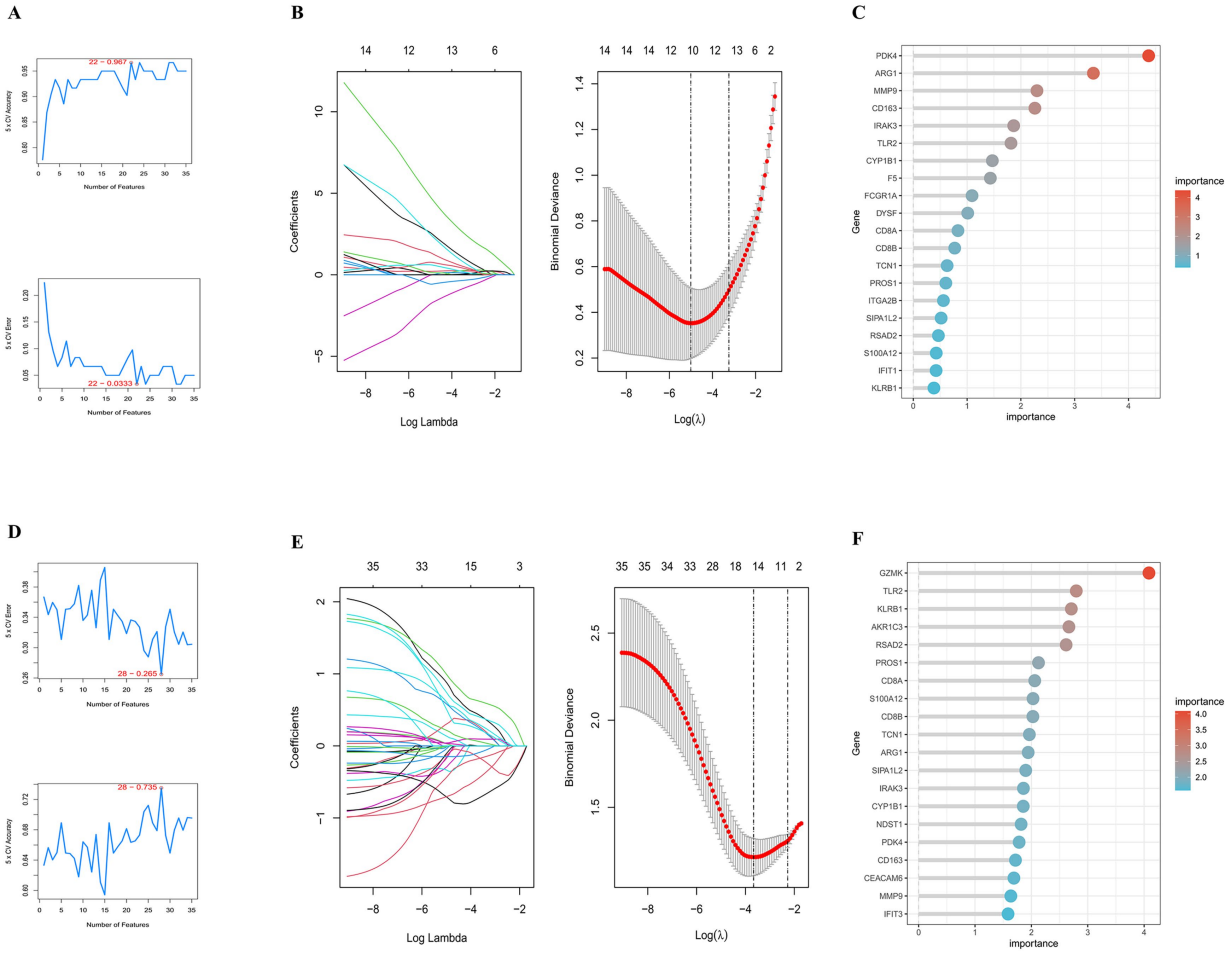
Identification of candidate core genes associated with PSD using three machine learning algorithms. **(A)** SVM-RFE analysis identified 22 genes in IS. **(B)** LASSO coefficient profile for IS demonstrating selection of the optimal tuning parameter *λ*. **(C)** Top 15 genes identified by RF analysis in IS ranked by variable importance. **(D)** Twenty-eight genes were identified through SVM-RFE analysis in MDD. **(E)** LASSO coefficient profile for MDD demonstrating selection of the optimal tuning parameter λ. **(F)** Top 15 genes identified by RF analysis in MDD ranked by variable importance.

To more precisely screen for hub genes with high diagnostic value, this study employed machine learning algorithms for feature gene selection. Using the preliminarily identified IDEGs, we sequentially conducted analyses using SVM-RFE, LASSO regression, and RF classification algorithms. The SVM-RFE algorithm identified 22 genes in the IS group with high diagnostic accuracy (0.967) with a low error rate (0.0333) (Figure 3A). In the MDD group, SVM-RFE identified 28 genes with an accuracy of 0.735 and an error rate of 0.265 (Figure 3D).

Subsequently, LASSO regression analysis, with the regularization parameter *γ* set to 0.006705562, screened 11 genes with non-zero coefficients in the IS group (Figure 3B). In the MDD group, setting γ to 0.02556914 identified 14 genes with non-zero coefficients (Figure 3E). Finally, the aforementioned IDEGs were input into the RF classifier, which determined the top 15 genes in both the IS and MDD groups (Figures 3C,F, respectively). Utilizing the “venny” tool to take intersections of the gene results obtained by different methods across different datasets ultimately identified two relevant core genes (TLR2 and CYP1B1) (Supplementary Figure 3).

### 3.4 Diagnostic value and validation of core biomarkers

**Figure 4:**
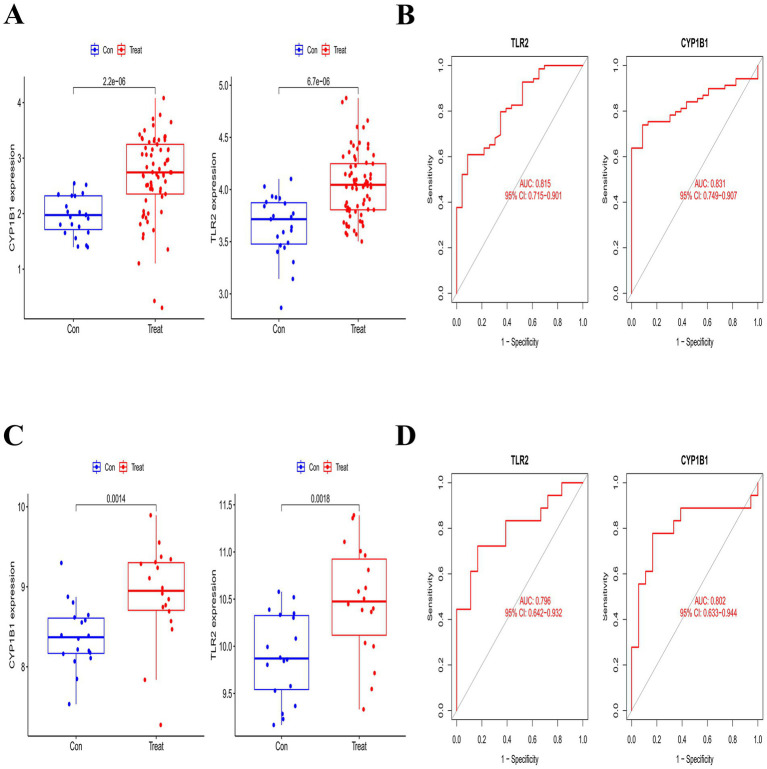
To assess the diagnostic performance of the two core genes, we utilized ROC curves to evaluate their diagnostic potential across different datasets. The expression levels of CYP1B1 and TLR2 were significantly altered compared to controls **(A,C)**. In the IS validation set (GSE58294), the AUC values for TLR2 and CYP1B1 were 0.815 and 0.831, respectively **(B)**. In the MDD validation set (GSE38206), the AUC values were 0.796 and 0.802, respectively **(D)**. These results indicate that both TLR2 and CYP1B1 demonstrated good diagnostic performance, suggesting their potential as diagnostic biomarkers for PSD.

To assess the diagnostic performance of the two core genes, we utilized ROC curves to evaluate their diagnostic potential across different datasets. The expression levels of CYP1B1 and TLR2 were significantly altered compared to controls (Figures 4A,C). In the IS validation set (GSE58294), the AUC values for TLR2 and CYP1B1 were 0.815 and 0.831, respectively (Figure 4B). In the MDD validation set (GSE38206), the AUC values were 0.796 and 0.802, respectively (Figure 4D). These results indicate that both TLR2 and CYP1B1 demonstrated good diagnostic performance, suggesting their potential as diagnostic biomarkers for PSD.

### 3.5 Immune cell infiltration and correlation with hub genes

**Figure 5:**
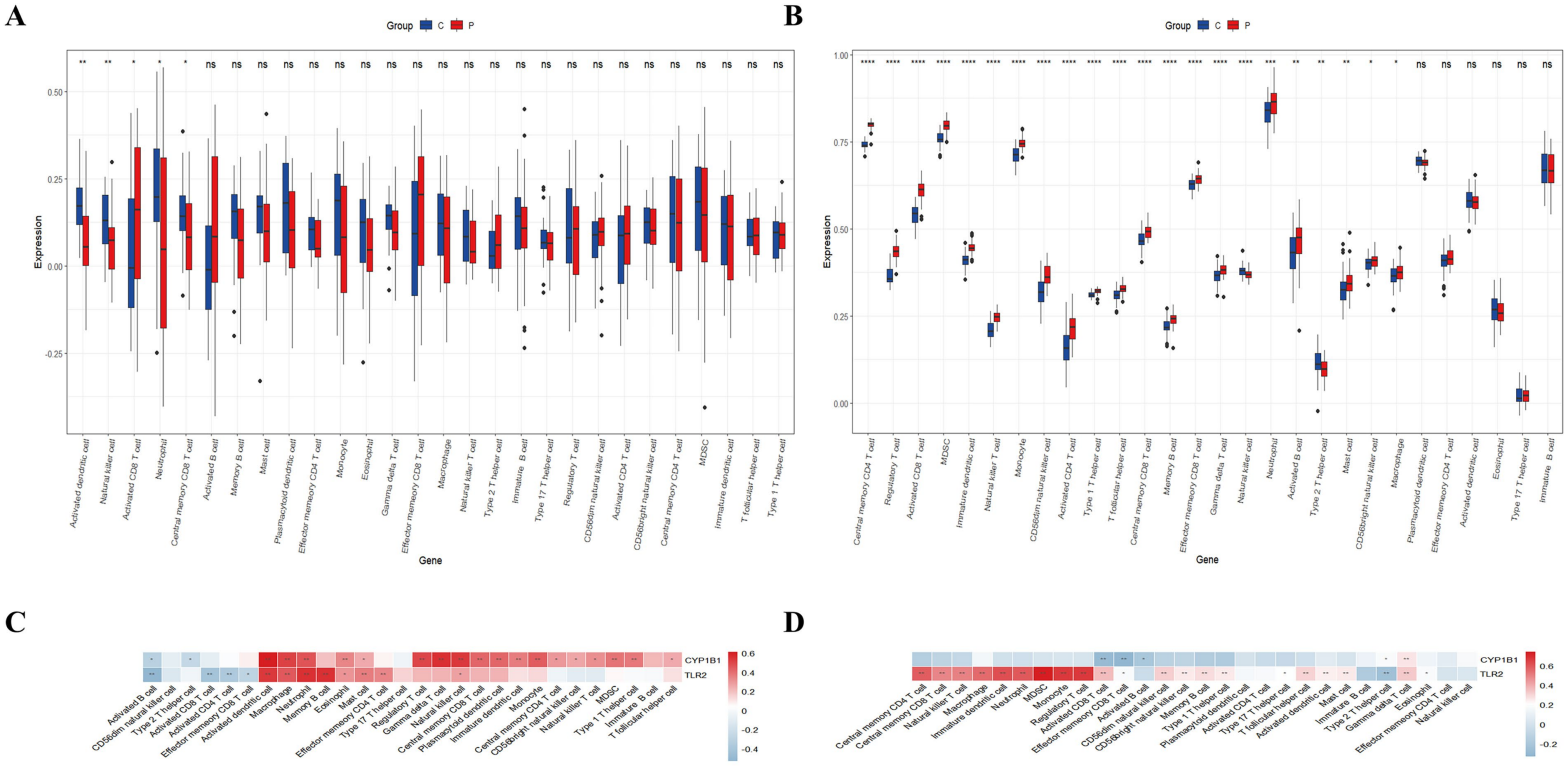
Immune cell composition in IS and MDD cohorts. **(A)** Distinct immune cell infiltration patterns in the IS group compared to controls. **(B)** Distinct immune cell infiltration profiles in the MDD group, with cell types exhibiting similar abundance trends to IS highlighted. **(C,D)** Correlation heatmaps presenting associations between CYP1B1 and TLR2 expression levels and immune cell subsets, respectively.

The ssGSEA method quantified 28 immune cell populations across MDD, IS, and control groups, revealing statistically significant variations in specific immunocyte subsets between disease states and healthy controls with consistent trends that were identified as potential immune cells, leading to the identification of two distinct immune cell types among the 28 assessed, including activated CD8 T cell and natural killer cell, where activated CD8 T cells were upregulated in the case groups while natural killer cells were downregulated (Figures 5A,B). Figures 5C,D illustrate the correlations between the core IDEGs and the immune cells, respectively.

### 3.6 Drug prediction and calculation of binding energy between drugs and core genes

**Figure 6:**
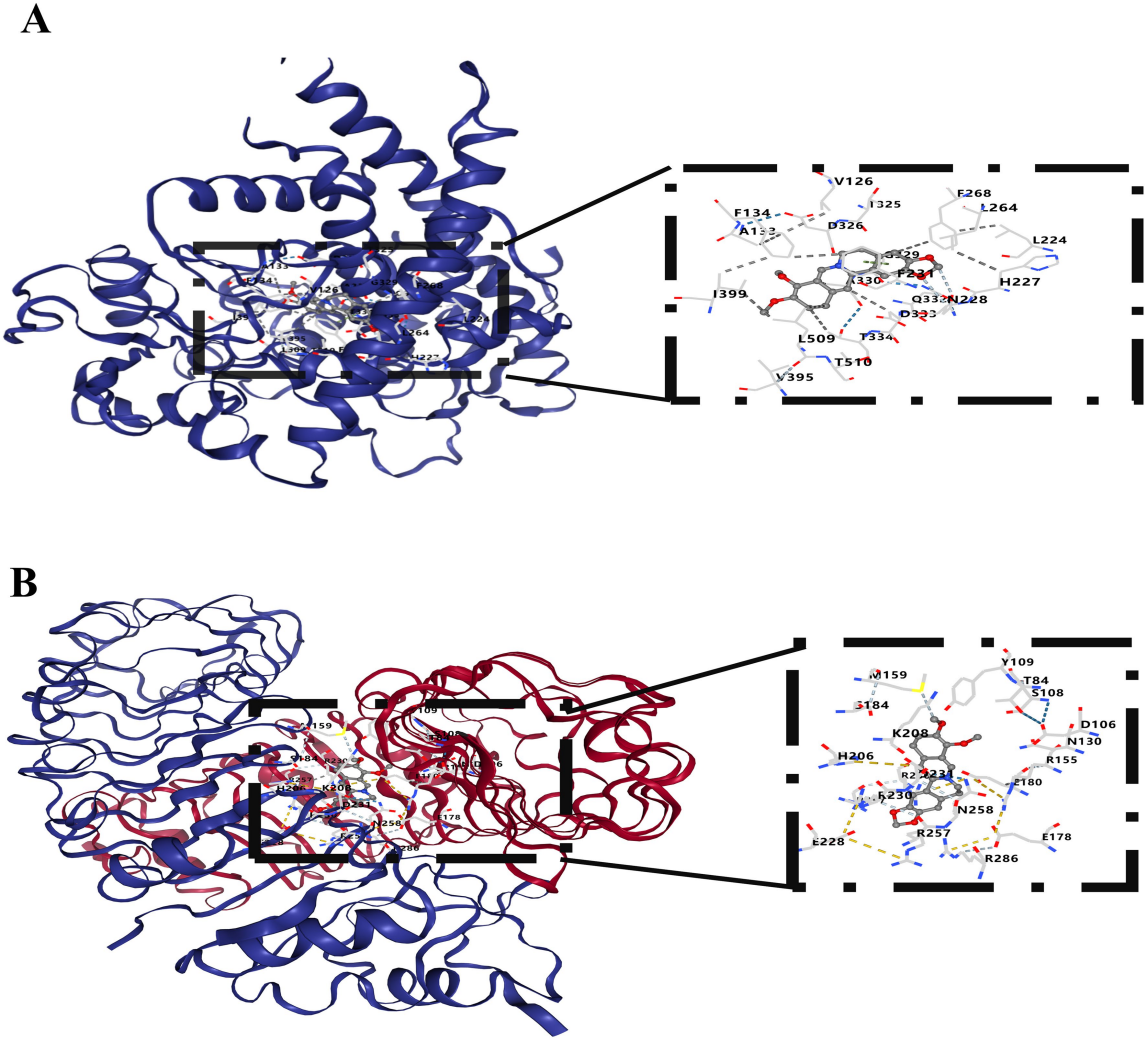
**(A,B)** The ligand binding sites within the protein and the interactions between the ligand and key residues. Docking poses were generated using structure-based blind docking. Potential binding sites for the ligand were ranked according to AutoDock Vina scores (kcal/mol), with the pose exhibiting the lowest binding energy selected for visualization.

To identify TCMs with potential therapeutic value, we screened the Coremine Medical database. A total of 17 TCMs related to these two core genes were obtained. Based on its documented efficacy in anti-inflammation, anti-cerebral ischemia, and anti-depression, Coptidis was selected as a potential therapeutic agent for PSD (Willner et al., 1987). Subsequently, 14 active compounds within Coptidis were retrieved from the TCMSP database. Based on previous research findings, the primary active ingredient of Coptidis, BBR (Mujtaba et al., 2022), was selected for further investigation. Molecular docking analysis was conducted using the CB-Dock2 online server. The results demonstrated that BBR, the active component of Coptidis, exhibited favorable binding energies with both TLR2 and CYP1B1. The binding energies of BBR with TLR2 and CYP1B1 were −8.2 kcal/mol and −10.2 kcal/mol, respectively (Figures 6A,B).

### 3.7 Evaluation of depression-like Behaviors

**Figure 7:**
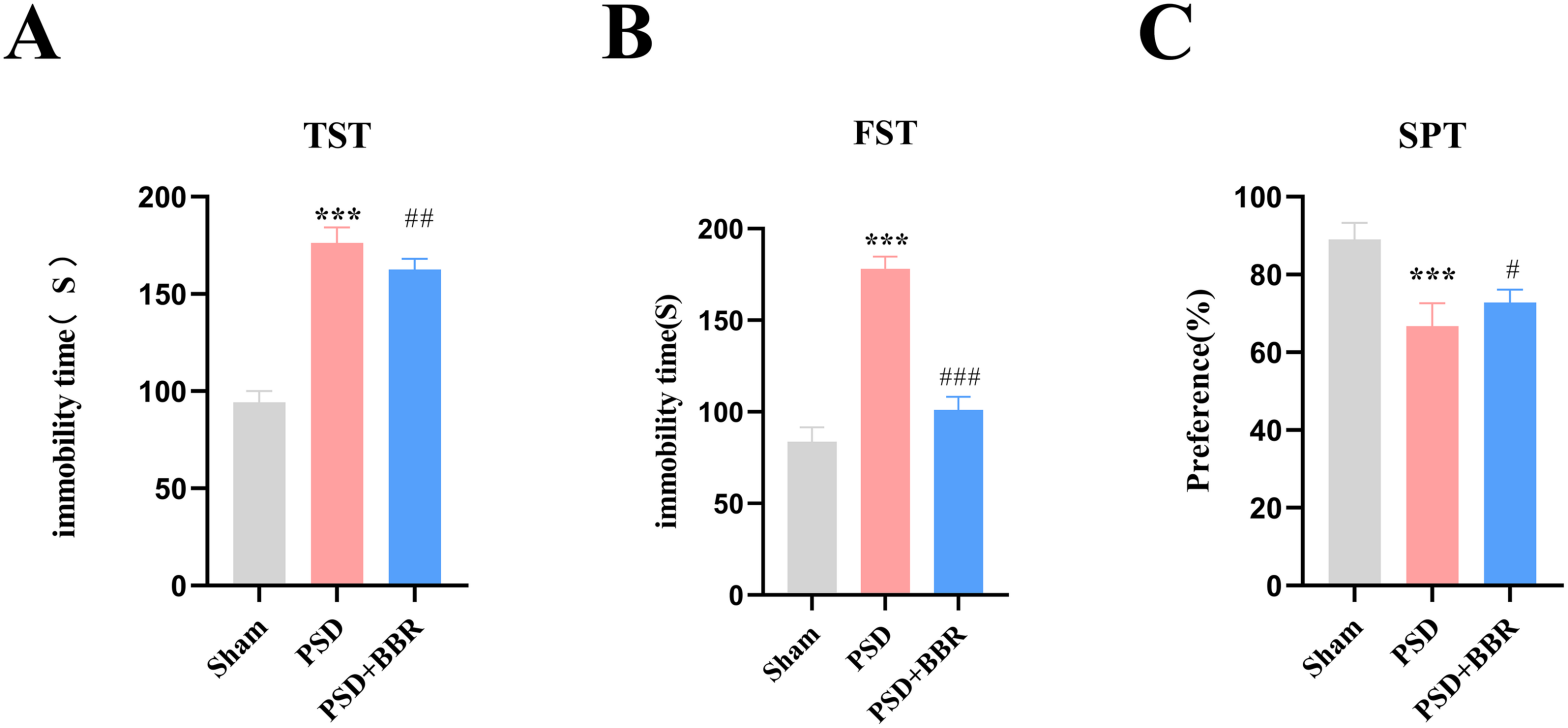
**(A)** Immobility duration of Sham, PSD, and PSD + BBR rats in the TST (*n* = 10 per group), evaluating the effects of PSD and BBR treatment. **(B)** Immobility duration in the FST among the three groups. **(C)** Sucrose preference in Sham, PSD, and PSD + BBR rats as assessed by the SPT. ***p < 0.001 Sham vs. PSD group; *#p* < 0.05, *##p* < 0.01, *###p* < 0.001 PSD vs. PSD + BBR group; one-way ANOVA followed by Tukey’s *post hoc* test; n = 10 rats per group.

Prior to the induction of depression-like behaviors, the MCAO model was validated by neurological deficit assessment using the Longa scoring system. Rats with scores ≥1 and <4 were considered successful MCAO models and were subsequently subjected to CUMS procedures to establish the PSD model. Behavioral assessments revealed more pronounced depressive-like behaviors in PSD rats. Rats subjected to PSD showed a marked reduction in climbing duration during the TST relative to the Sham controls (Figures 7A,B). Conversely, rats treated with BBR showed significantly increased climbing time and reduced immobility time compared to the PSD group. These findings demonstrate BBR’s antidepressant effects in PSD models. In the sucrose preference test (SPT), PSD rats displayed a significant reduction in sucrose preference. Oral administration of BBR to PSD rats significantly improved this depressive-like behavior (Figure 7C)

### 3.8 Reduced neuronal density in the hippocampal CA1 region of PSD rats

**Figure 8:**
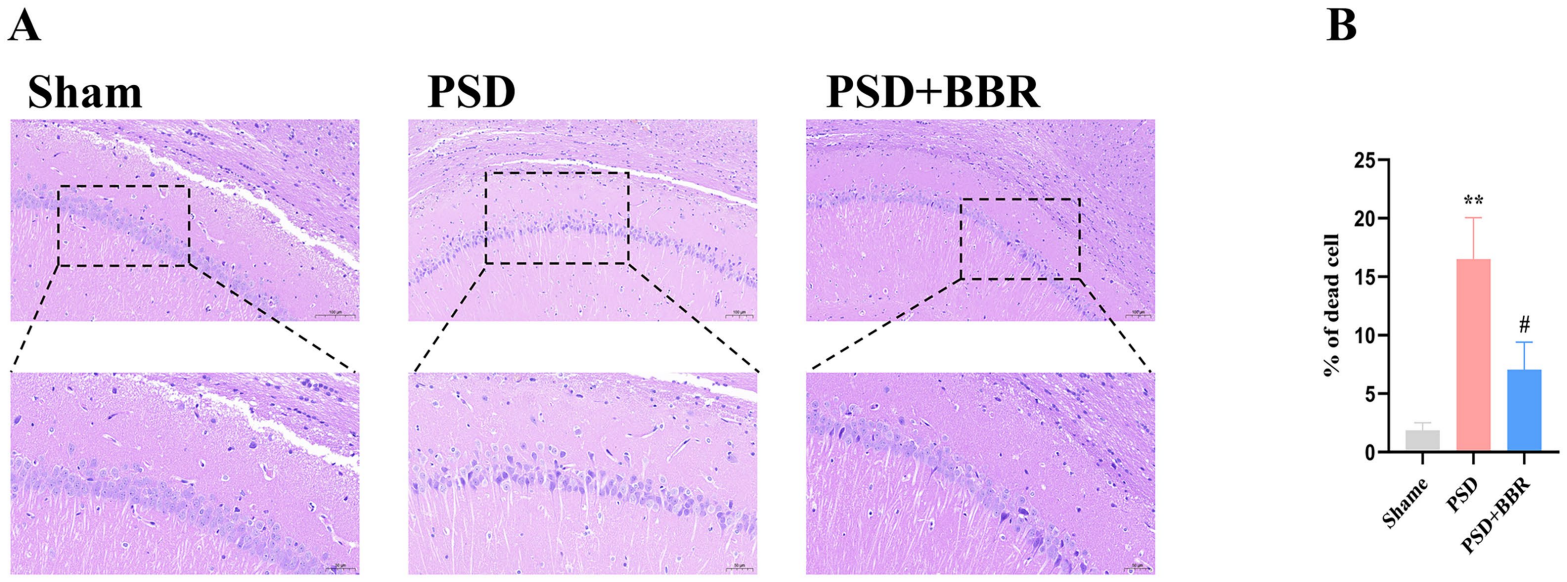
Histopathological changes in the hippocampal CA1 region of rats. **(A)** Representative hematoxylin and eosin (HE) staining images of the hippocampal CA1 region in the Sham, PSD, and PSD + BBR groups, respectively. The PSD group showed neuronal loss, nuclear pyknosis, and disordered cellular arrangement compared with the Sham group, while BBR treatment partially alleviated these pathological changes. **(B)** Quantitative analysis of neuronal death rate in the hippocampal CA1 region. Data are expressed as mean ± SEM (*n* = 10 per group). **p < 0.01 Sham vs. PSD group; #p < 0.05 PSD vs. PSD + BBR group; one-way ANOVA followed by Tukey’s *post hoc* test.

HE staining was performed to assess neuronal morphological alterations in PSD rats and evaluate the neuroprotective effects of BBR treatment. HE staining results demonstrated that neurons in the Sham group were well-arranged with intact morphology and no apparent structural abnormalities. In stark contrast, neurons in the PSD group exhibited a disordered arrangement, significant neuronal loss, nuclear condensation, increased basophilia, and cellular atrophy or necrosis, indicating substantial neuronal damage induced by PSD. However, in the PSD + BBR treatment group, neuronal organization was improved, and neuronal damage was significantly mitigated (Figure 8A). Quantitative analysis confirmed these observations. The neuronal death rate in the CA1 region was significantly increased in the PSD group compared with the Sham group. Treatment with BBR markedly reduced the neuronal death rate compared with the PSD group, although it did not fully restore it to the Sham group (Figure 8B). These findings provide quantitative evidence supporting the neuroprotective effect of BBR.

### 3.9 Elevated inflammatory response in PSD rats

**Figure 9:**
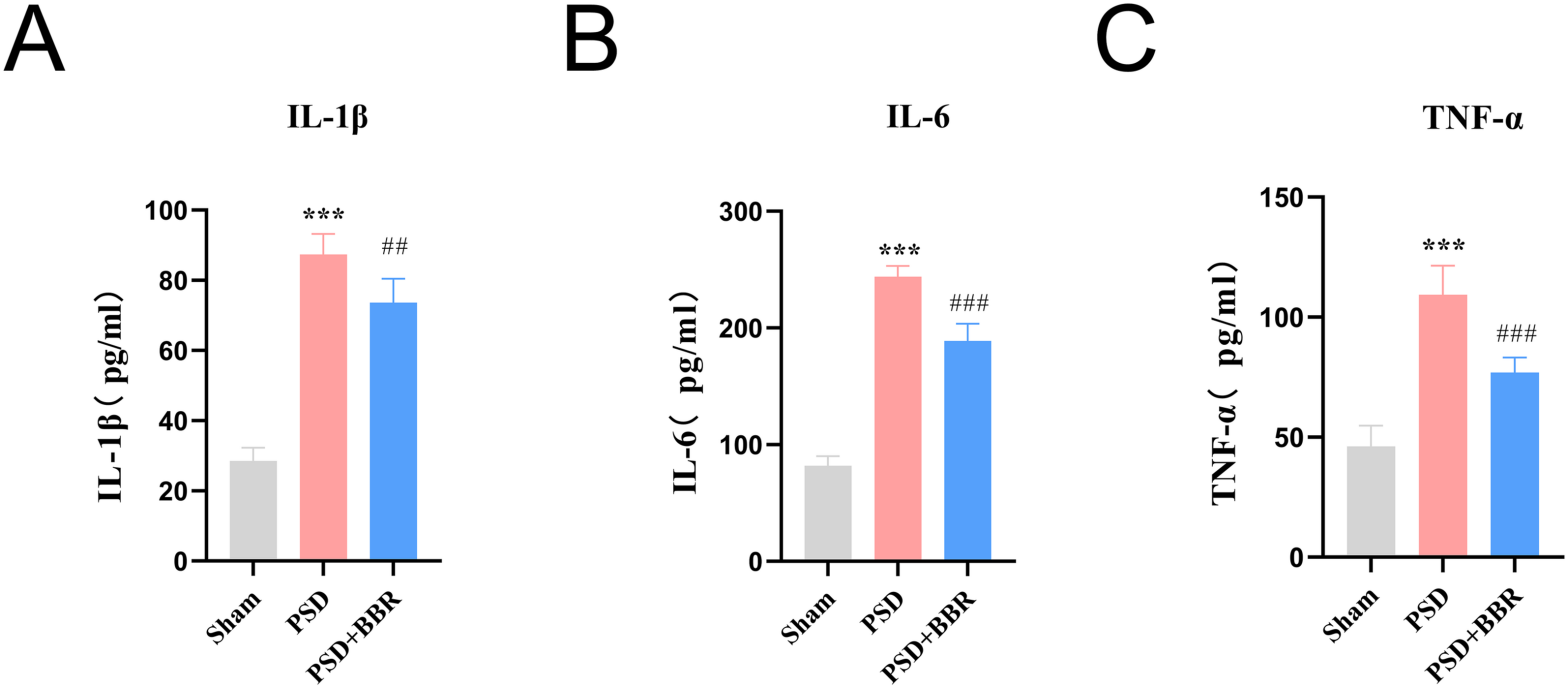
Inflammatory response in rats with PSD. **(A)** ELISA quantification of the pro-inflammatory cytokine IL-1β. **(B)** ELISA quantification of the pro-inflammatory cytokine IL-6. **(C)** ELISA quantification of the pro-inflammatory cytokine TNF-α. ***p < 0.001, Sham vs. PSD group, ##*p* < 0.01, ###*p* < 0.001, PSD vs. PSD + BBR group.

To validate the expression of inflammatory factors in PSD, we measured the levels of IL-1β, IL-6, and TNF-*α* in rat brain tissue. The results revealed that compared with the Sham group, rats in the PSD group exhibited significantly elevated levels of IL-1β, IL-6, and TNF-α. In contrast, the PSD + BBR group showed significantly reduced levels of these inflammatory cytokines (Figures 9A–C). These findings suggest that BBR inhibits the inflammatory response in PSD rats.

### 3.10 Increased TLR2 and CYP1B1 protein expression in rat brain tissue post-PSD

**Figure 10:**
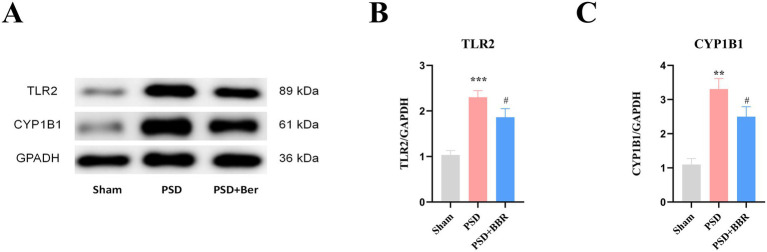
Protein expression levels of TLR2 and CYP1B1 in the PSD rat model. **(A)** Representative Western blot images displaying protein bands for TLR2 and CYP1B1. **(B)** Quantification of TLR2 band intensity normalized to GAPDH. **(C)** Quantification of CYP1B1 band intensity normalized to GAPDH. **p < 0.01, ***p < 0.001, Sham vs. PSD group, #*p* < 0.05, PSD vs. PSD + BBR group.

To investigate the expression of TLR2 and CYP1B1 in PSD, we detected their protein levels in rat brain tissue using Western blot analysis. The results revealed that TLR2 and CYP1B1 expression were significantly elevated in the PSD group compared with the Sham group, which is consistent with our bioinformatics analysis. Conversely, expression levels of both proteins were markedly reduced in the PSD + BBR group relative to the PSD group (Figures 10B,C). These results imply that BBR may exert a regulatory effect on TLR2 and CYP1B1 during the pathogenesis of PSD (Figure 10).

## 4 Discussion

Stroke imposes a significant burden on public health systems and family economies due to its high incidence and disability rate. PSD represents both the most common post-stroke neurobehavioral disorder and an autonomous mortality predictor in cerebrovascular patients (Sunhe et al., 2024).

However, the underlying pathogenesis of PSD remains incompletely elucidated, and the lack of well-defined diagnostic criteria severely hinders its early identification and intervention. Consequently, exploring the pathogenesis of PSD and identifying early therapeutic strategies are crucial. In this study, we combined bioinformatics approaches utilizing microarray datasets from the GEO database to identify IDEGs and their associated molecular pathways core to the pathogenesis of PSD (Liu et al., 2024). Functional annotation of IDEGs highlighted predominant enrichment in inflammatory processes, autophagic regulation, and immune-related signaling pathways (Rap1, IL-17, T-cell receptor), corroborating the established neuroinflammatory basis of PSD (D'Anci et al., 2019; Sales et al., 2021; Yang et al., 2023). Through integrated machine learning approaches (SVM-RFE, LASSO, RF), we identified TLR2 and CYP1B1 as key biomarkers, with ROC validation confirming their diagnostic utility for PSD detection.

Same as previous research (Kim et al., 2024; Tajalli-Nezhad et al., 2019; D'Uva et al., 2018), our results strongly suggest that TLR2 and CYP1B1 may contribute to PSD development, potentially by mediating inflammatory cascades. Molecular docking simulations further indicated that BBR exhibited favorable binding affinity and potential interaction sites with both TLR2 and CYP1B1. This prediction was experimentally validated by our *in vitro* findings, confirming the therapeutic mechanism of BBR in PSD.

PSD represents a heterogeneous disorder, as no single pathophysiological mechanism fully accounts for its development. Emerging research implicates neuroinflammatory pathways in the pathogenesis of PSD (Hu et al., 2019), where post-stroke immune activation mediates elevated cerebral cytokine production (IL-1β, IL-6, TNF-*α*).

These cytokines can directly impair neuronal synaptic function (Das and Rajanikant, 2018), and they can also induce alterations in cerebral neurotransmitters, reduce neuroplasticity through sustained inflammatory cascades, and ultimately lead to depressive symptoms characterized by mood, cognitive, and behavioral changes (Fan et al., 2023). TLR2, a pivotal immune receptor, recognizes diverse pathogen-associated molecular patterns (PAMPs) and DAMPs. Its activation potently stimulates downstream signaling pathways, especially the NF-κB pathway, resulting in the excessive production of pro-inflammatory cytokines and the establishment of a chronically detrimental inflammatory microenvironment within the brain (Ma et al., 2024; Dutta et al., 2023; Falero-Perez et al., 2019). Concurrently, CYP1B1, a cytochrome P450 enzyme, generates bioactive metabolites with immunomodulatory activity (Abarca-Merlin et al., 2024). CYP1B1 overexpression is associated with the production of pro-inflammatory metabolites in various pathological contexts, which exacerbate inflammatory responses (Han et al., 2023; Sun et al., 2023; Singh et al., 2020; Zhang et al., 2010). To validate the role of this pathway in PSD, we established a rat model of PSD. MCAO validation using the Longa scoring system was performed prior to CUMS exposure, ensuring that the observed depressive-like phenotypes were based on consistent ischemic injury backgrounds. Our findings demonstrated that TLR2 and CYP1B1 overexpression induced depressive-like behaviors in post-stroke rats. Besides, we observed significant neuronal loss and markedly elevated inflammatory cytokine expression in the brain tissue of the PSD group. Based on these collective results, we propose that TLR2 and CYP1B1 play crucial roles in the pathogenesis of PSD, with their expression levels strongly correlating with the intensity of the inflammatory response and the extent of neuronal damage. These findings further substantiate the contribution of neuroinflammation to PSD and highlight TLR2 and CYP1B1 as potential biomarkers for this condition.

Based on these key findings, we further examined the therapeutic potential of BBR in PSD. BBR possesses demonstrated neuroprotective and anti-inflammatory properties in ischemic brain injury (Jiang et al., 2024; Roh et al., 2020). Research indicates that BBR effectively suppresses TLR2-mediated signaling pathways, thereby suppressing downstream inflammatory mediators, including IL-1β and TNF-α (Zhang et al., 2012).

Although our experimental analyses were performed on whole-brain tissue, we acknowledge that depression-related pathophysiology is closely linked to specific brain nuclei such as the hippocampus, prefrontal cortex, and amygdala. Previous studies have demonstrated that inflammatory signaling in the hippocampus can impair neurogenesis and synaptic plasticity (Gulyaeva, 2019a). In the present study, our HE staining of the hippocampal CA1 region already indicated neuronal loss and pathological changes in PSD rats, which were partially reversed by BBR treatment. This finding is consistent with the established role of the hippocampus in depression. Future work will focus on dissecting region-specific contributions of TLR2- and CYP1B1-mediated neuroinflammatory processes within these nuclei, which may provide more precise mechanistic insights and therapeutic targets.

Furthermore, emerging evidence suggests that BBR can inhibit CYP1B1 expression (Kumar et al., 2024), and the potential mechanism may be linked to its anti-inflammatory effects (ElKhatib et al., 2024). Similar to these previous findings, our experimental data demonstrate that BBR ameliorated depressive-like behaviors and attenuated neuronal damage in PSD rats. Concomitantly, BBR treatment effectively suppressed the overexpression of TLR2 and CYP1B1 and ameliorated systemic inflammation in PSD rats. Collectively, these results indicate that BBR may alleviate depressive symptoms and preserve neuronal integrity in PSD rats by suppressing the expression of both TLR2 and CYP1B1, thereby inhibiting neuroinflammation.

In summary, this investigation clarifies the pathogenesis of PSD, identifying TLR2 and CYP1B1 as promising diagnostic markers and treatment targets. The Coptidis alkaloid BBR demonstrates therapeutic potential against PSD, likely by suppressing the overexpression of TLR2 and CYP1B1. This suppression inhibits downstream inflammatory cascades, reduces neuronal damage, and ultimately improves behavioral outcomes in PSD. Furthermore, our findings reveal the critical link between neuroinflammation and PSD, suggesting that neuroinflammatory mechanisms play a crucial role in PSD development following cerebrovascular events.

Regarding the ability of BBR to exert central effects, recent studies have confirmed that BBR is capable of crossing the blood–brain barrier (BBB), although with relatively low permeability, and accumulates in brain tissue after systemic administration (Mehboodi et al., 2024). This property supports the plausibility of our observed central antidepressant and neuroprotective effects in the PSD model. Therefore, BBR targeting TLR2 and CYP1B1 to modulate neuroinflammation may represent a promising therapeutic strategy for PSD. Nonetheless, different dosages or optimized delivery strategies (e.g., nanoparticle carriers or intranasal administration) may further improve brain bioavailability and potentially enhance therapeutic efficacy. Future studies are warranted to systematically evaluate dose–response relationships and alternative administration routes.

## 5 Conclusion

This research advances understanding of the pathogenesis of PSD while proposing innovative diagnostic markers and intervention strategies for improved clinical management.

Specifically, the identified roles of TLR2 and CYP1B1 in the pathogenesis of PSD may offer new avenues for clinical management. Furthermore, as a key bioactive constituent of Coptidis, BBR demonstrates promising therapeutic potential against PSD and warrants further investigation. Certain limitations of this study should be acknowledged, including the need for validation through larger clinical cohorts to fully establish the translational significance of these findings.

## Data Availability

Publicly available datasets were analyzed in this study. This data can be found here: GSE16561, GSE98793, GSE38206, and GSE58294.

## Footnotes

^1^ https://www.ncbi.nlm.nih.gov/geo/

^2^ www.genecards.org

^3^ http://www.sangerbox.com/tool

^4^ https://www.coremine.com/medical/

^5^ https://tcmsp-e.com/tcmsp.php

^6^ https://www.rcsb.org/

^7^ https://cadd.labshare.cn/cb-dock2/
