# Supplementary material for: Inflammation-related biomarkers and berberine therapy in post-stroke depression: evidence from bioinformatics, machine learning, and experimental validation
Source: Front Neurosci. 2025 Oct 14;19:1684297. doi: 10.3389/fnins.2025.1684297 (PMC12558837; doi:10.3389/fnins.2025.1684297)
Supplement: Supplementary file 1 [file Data_Sheet_1.DOCX]

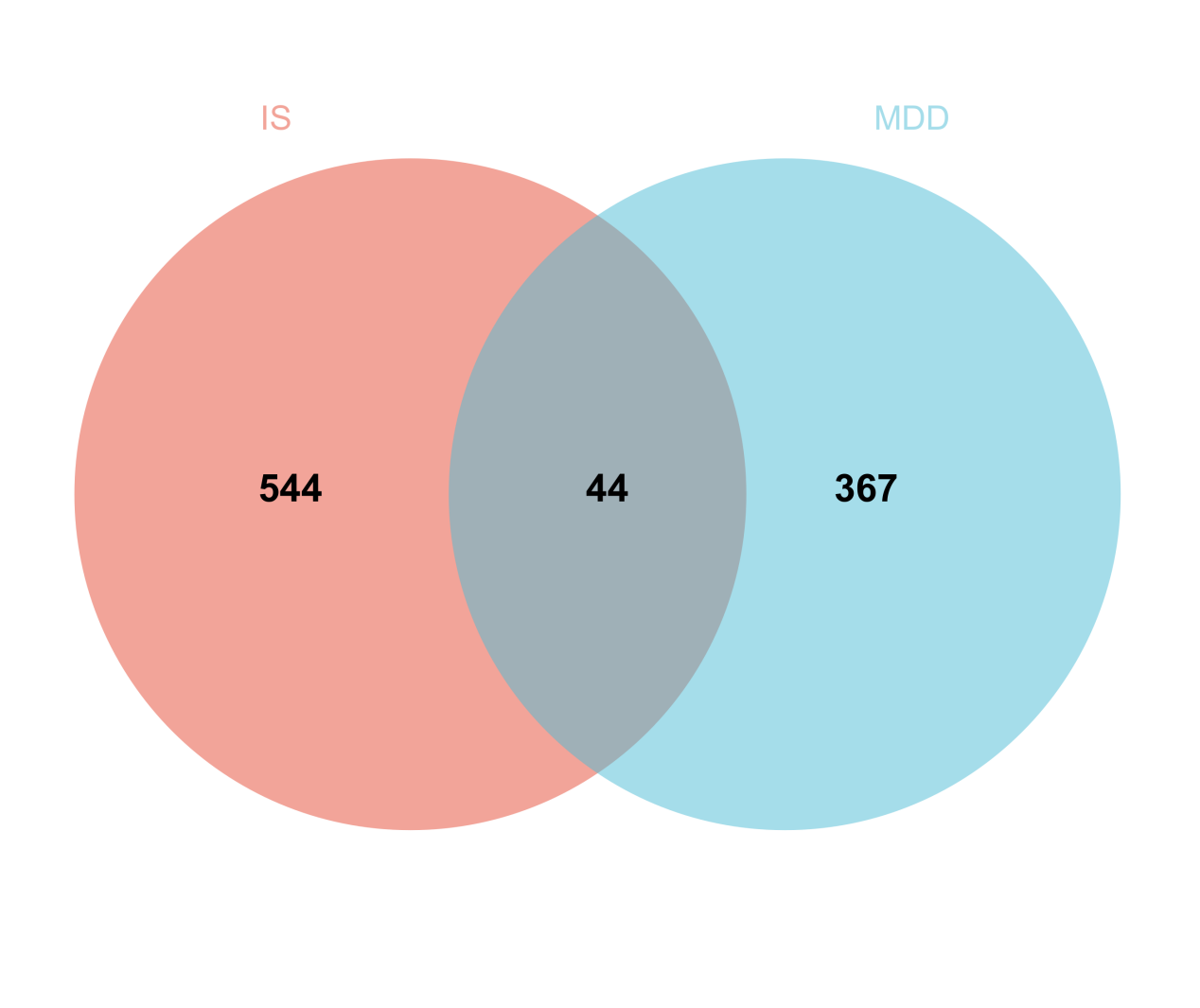


**Supplementary Figure 1:** Venn diagram of PSD-related differentially expressed genes (DEGs). The diagram illustrates the overlap of DEGs identified in the IS dataset (GSE16561) and the MDD dataset (GSE98793), showing DEGs potentially involved in PSD.


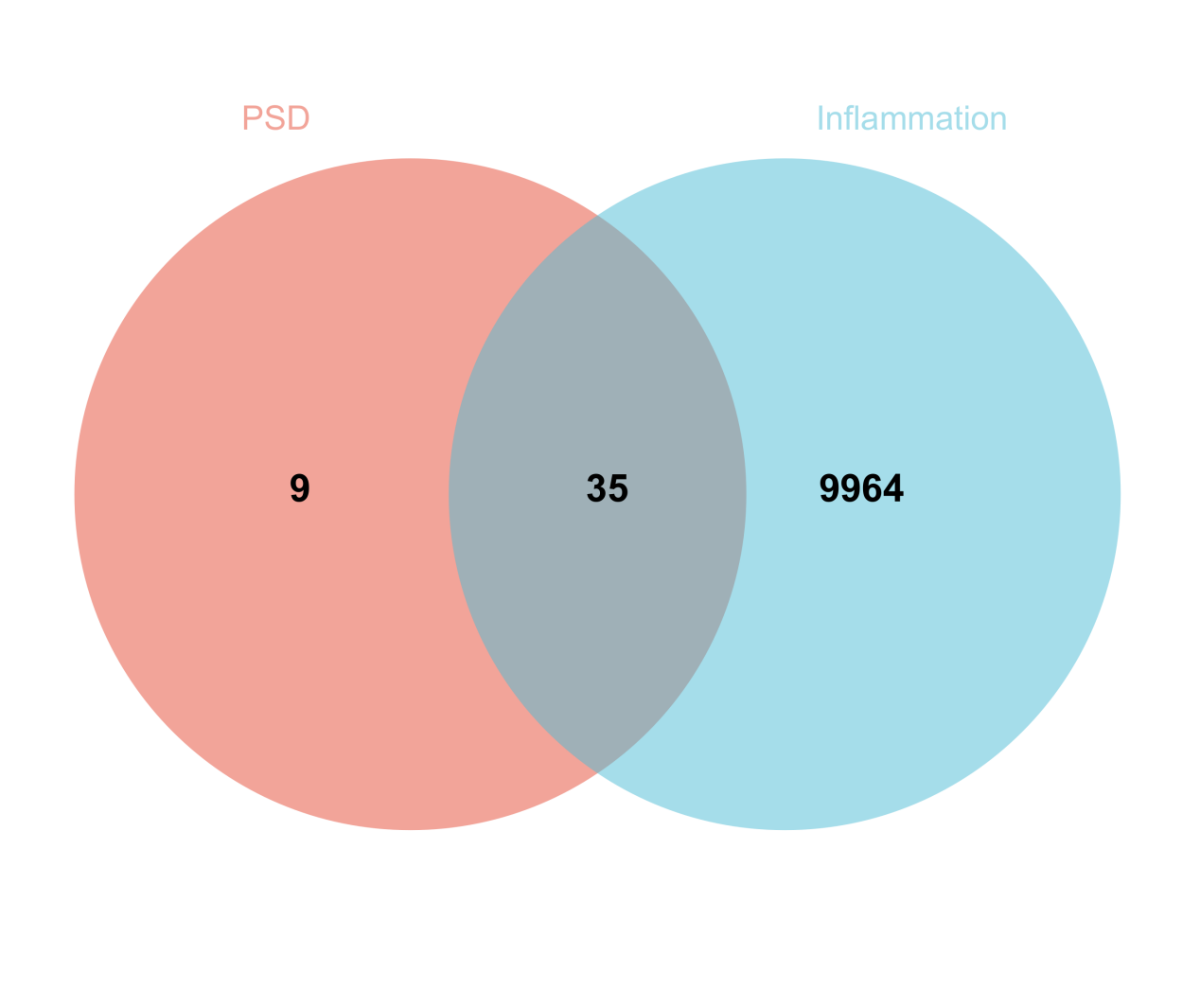


**Supplementary Figure 2:**Venn diagram of inflammation-related genes associated with PSD. The diagram highlights inflammation-related genes potentially implicated in PSD pathogenesis.


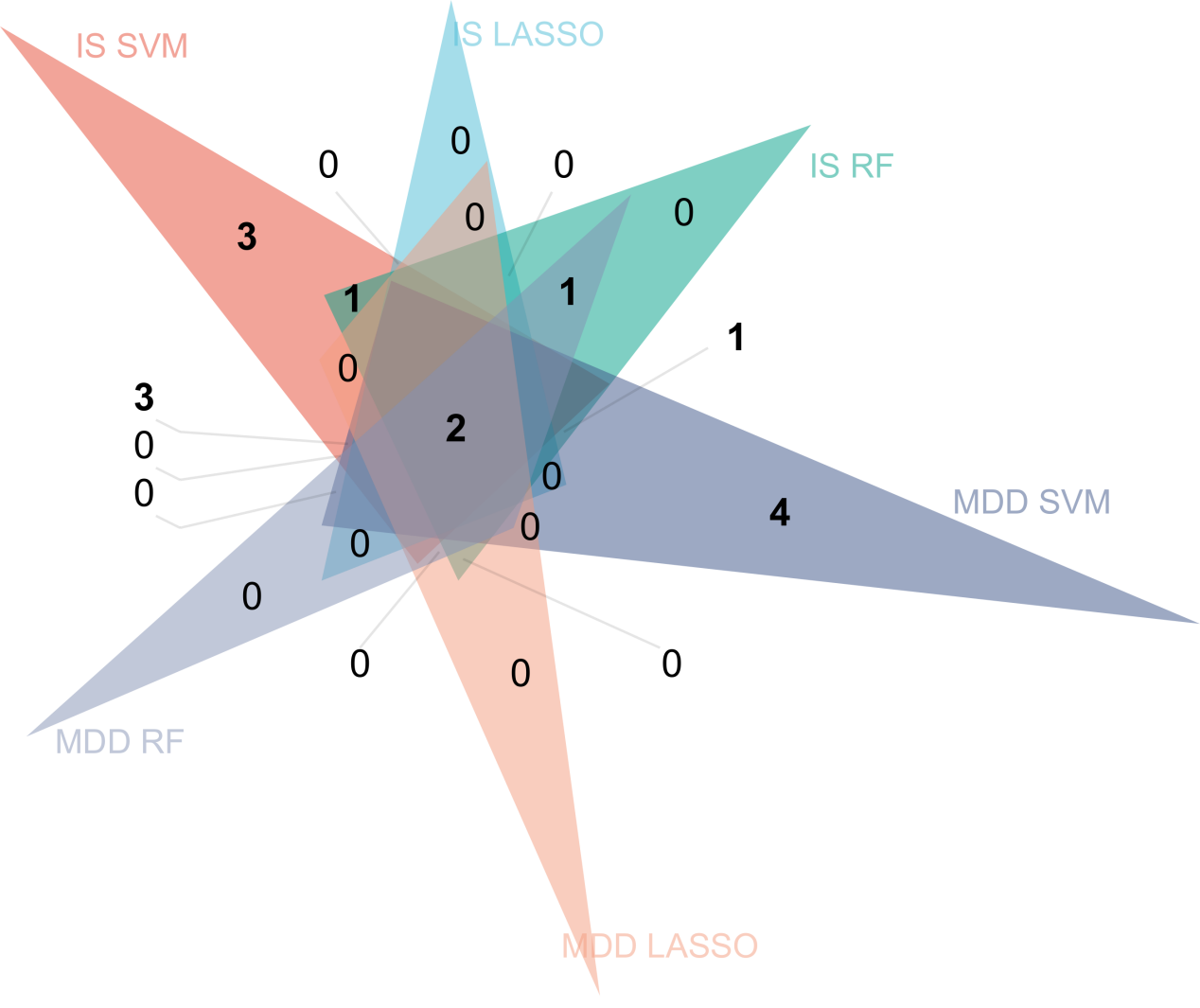


**Supplementary Figure 3:**Venn diagram illustrating the intersection of candidate genes identified by three machine-learning algorithms (LASSO, SVM-RFE, and Random Forest) across datasets. The overlapping region highlights genes consistently selected by multiple methods, representing robust PSD-related biomarkers.
